# Molecular Diagnosis as an Alternative for Public Health Surveillance of Leptospirosis in Colombia

**DOI:** 10.3390/microorganisms11112759

**Published:** 2023-11-13

**Authors:** Margarita Arboleda, Mariana Mejía-Torres, Maritza Posada, Nicaela Restrepo, Paola Ríos-Tapias, Luis Alberto Rivera-Pedroza, David Calle, Miryan M. Sánchez-Jiménez, Katerine Marín, Piedad Agudelo-Flórez

**Affiliations:** 1 Tropical Medicine Group, Colombian Institute of Tropical Medicine, Sabaneta 055450, Colombia; mmejiat@ces.edu.co (M.M.-T.); maritza.posada@udea.edu.co (M.P.); nicaela.restrepo@udea.edu.co (N.R.); parios@ces.edu.co (P.R.-T.); lriverap@ces.edu.co (L.A.R.-P.); dcalle@ces.edu.co (D.C.); msanchez@ces.edu.co (M.M.S.-J.); kmarin@ces.edu.co (K.M.); 2 Graduate School, Universidad CES, Medellín 050021, Colombia

**Keywords:** leptospirosis, diagnosis, PCR, Colombia, public health surveillance

## Abstract

Leptospirosis represents a public health problem in Colombia. However, the underreporting of the disease is an unfortunate reality, with a clear trend towards a decrease in cases since 2019, when the guidelines for its confirmatory diagnosis changed with the requirement of two paired samples. The purpose of this review is to highlight the importance of leptospirosis. While the access to rapid diagnosis is available at practically all levels of care for dengue and malaria, leptospirosis—a doubly neglected disease—deserves recognition as a serious public health problem in Colombia. In this manner, it is proposed that molecular tests are a viable diagnostic alternative that can improve the targeted treatment of the patient and the timeliness of data and case reporting to SIVIGILA, and reduce the underreporting of the disease. Taking advantage of the strengthened technological infrastructure derived from the SARS-CoV-2 pandemic for molecular diagnosis in Colombia, with a network of 227 laboratories distributed throughout the national territory, with an installed capacity for PCR testing, it is proposed that molecular diagnosis can be used as an alternative for early diagnosis. This would allow case confirmation through the public health network in Colombia, and, together with the microagglutination (MAT) technique, the epidemiological surveillance of this disease in this country would be strengthened.

## 1. Introduction

Leptospirosis is a zoonotic disease caused by bacteria of the genus *Leptospira*, belonging to the phylum Spirochaetes, which represents a public health problem worldwide [1,2]. This disease, which affects humans and other animals, is considered an endemic disease mainly of tropical and subtropical climates [1,3,4]. There has also been a clear increase in registered cases in temperate climate regions in recent years due to certain factors [1,5]. These include climate change, migrations–particularly of travelers returning from tropical areas [6]–overcrowding, poor waste disposal and inadequate solid waste management [5,7,8,9], and an increase in the rodent population, as in the case of the reemergence of leptospirosis in New York City [10,11].

Leptospirosis transmission has been associated with socioeconomic conditions, contact with both animal and water reservoirs, inhabitants of urban slums, high-risk occupations such as farmer, veterinarian, miner, and sewer cleaning worker [5,12]. Additionally, freshwater recreational activities, socio-cultural globalization, climate change, tropical storms, hurricanes, and migrations of both humans and animals to endemic areas are recognized as risk factors favoring its transmission [7,13]. The most vulnerable populations are those living in areas far from medical care and with low resources, where rapid diagnosis, and therefore access to adequate and timely treatment, is difficult [14,15].

The various pathogenic species of *Leptospira* spp. can infect numerous animal hosts including rodents, canines, cattle, swine, and other domestic animals, while humans are considered incidental hosts [3,12,16]. Domestic and wild animals carrying *Leptospira* spp. Can shed the bacterium in their urine for several years or a lifetime [3,16,17]. Its infection cycle in humans begins with the entry of spirochetes through mucous membranes or skin wounds by direct contact with the urine of infected animals, or indirectly with contaminated and/or stagnant water [12,18]. The pathogenic species then optimize their metabolism according to the host’s body temperature, evade the immune response when it is present [19], and generate non-specific symptoms such as fever, headache, and joint and musculoskeletal pains [20,21,22] that may simulate other infectious diseases such as dengue [20,21,22,23], malaria [20,21,22], influenza [12], Zika virus [20], and Hantavirus [21,24]. In severe cases, there may be meningeal involvement, jaundice, renal failure, and/or hemorrhage, which characterize Weil’s disease [25,26], and respiratory compromise such as severe pulmonary hemorrhage syndrome [27], or other uncommon manifestations [28].

Leptospirosis is often considered a doubly neglected disease [14] and, despite the availability of various diagnostic methods, the persistent problem of the underreporting of cases remains a major concern [29]. This problem is attributed, in part, to the absence of a comprehensive diagnostic approach that would facilitate notification to public health surveillance entities. The main objective of this review is to advocate for the adoption of a molecular diagnostic approach for leptospirosis in Colombia. This proposal is based on a comprehensive evaluation and comparison of the various diagnostic methods used for this disease, in order to put an end to the persistent problem of widespread underreporting in the country over the years. In addition, this proposal is supported by the improvement of laboratory infrastructures and the presence of trained personnel, resources acquired in response to the requirements of the COVID-19 pandemic, thus recognizing its potential to accelerate the integration of molecular tests as a diagnostic method.

## 2. Disease Burden and Underreporting

The World Health Organization (WHO) estimates the presence of 500,000 annual cases of leptospirosis in the world, most of them with a severe manifestation, for which it is estimated that mortality is higher than 10% [4,27]. Due to underreporting and misdiagnosis, estimating the number of human cases worldwide is challenging [29]. It has been estimated that there are 1.03 million cases worldwide and 58,900 deaths annually [4]. The tropical regions of South and Southeast Asia, the Western Pacific, Africa, and South and Central America are the areas with the highest estimated burden of leptospirosis globally. The five South American countries most affected by this disease according to the calculation of years of life lost (YLL) are Trinidad and Tobago, Guyana, Ecuador, Suriname, and Colombia [30].

A 2014 study on the incidence of leptospirosis in Latin America found that 19 of 21 countries included in the study reported a total of 10,088 human cases out of a population of approximately 610.9 million inhabitants. The countries with the highest recorded number of cases were Brazil (40.2%), followed by Peru (23.6%), Colombia (8.8%), and Ecuador (7.2%). The cumulative incidence rate for Latin America was 2.0 cases per 100,000 population, and males were afflicted in 65.1% of cases [31]. It is recognized that the importance of the disease on a global scale is increasing [32].

## 3. Diagnosis

The early diagnosis of the disease should be made within the first five days after the onset of symptoms and is usually based on at least two different techniques, either at the same time or in succession [33,34,35]. In turn, early diagnosis allows treatment to be more effective when given in the acute phase of the disease [36]. Between 5 and 10% of patients with leptospirosis may potentially develop a severe form [22], with a mortality rate of more than 10% due to Weil’s disease [37,38,39]. Leptospirosis is a disease the mortality of which is considered avoidable under epidemiological surveillance, contingent upon cases being caught early and treated in a timely manner [40,41].

The reference test for the diagnosis of leptospirosis is the detection of antibodies by microagglutination (MAT) [42]. Leptospirosis culture, polymerase chain reaction (PCR) and histopathological examination (preferably kidney, liver, lung, heart, or spleen tissues) are also considered reference tests for the diagnosis of the disease, since they demonstrate the presence of the microorganism [12,43,44].

### 3.1. Microscopic Examination

Although there are direct tests, such as dark field microscopy, that allow for the visualization of circulating leptospires in the direct examination of whole blood or urine in the acute phase when they reach a concentration above 104 leptospires per mL [45], the presence of fibrin threads in blood and some serum proteins or cellular debris can be confused with spirochetes, yielding false positives (sensitivity 40.2%, specificity 61.5%) [46]. This reduces the reliability of the test; thus, its low accuracy prevents its use as a diagnostic method [47,48], rendering this test valid only for checking *Leptospira* spp. cultures [45].

### 3.2. Culture

Culture from blood in the acute phase—the first 10 days of the disease—as well as urine, cerebrospinal fluid when there is neurological involvement, or tissues in post mortem cases, can be performed in special media such as Fletcher or Ellinghausen–McCullough–Johnson–Harris (EMJH) [45,49]. Generally, growth is slow [49] and should be followed for at least 8 weeks or more before a negative result can be issued [50].

### 3.3. Enzyme-Linked Immunosorbent Assay

The detection of antibodies (Ac) of the immunoglobulin M (IgM) type for *Leptospira* is usually possible using serological tests from the fifth or seventh day of symptom onset and can persist for 3 months (though there are reports of up to 9 months) [51], making it difficult to identify acute cases [52]. This indirect ELISA (enzyme-linked immunosorbent assay) test uses an antigen derived from *Leptospira* interrogans serovar Hardjo, which coats strips of reactive wells with antigen. Then, the specific antibodies present in the patient’s sample are bound to these solid phase antigens; this test is qualitative and is the standard technique for leptospirosis diagnosis in Colombia with Pan-Bio reagents [43].

### 3.4. Indirect Immunofluorescence

Immunofluorescence (IFI) stands out as a simple execution and interpretation technique, especially when compared to the microscopic agglutination test (MAT). Unlike MAT, it does not involve the handling of live bacteria in the execution phase and in the reading of the results. It also has the advantage of detecting the presence of IgM antibodies, which are usually present in the acute phase of the disease. It can be performed in laboratories that have an ultraviolet light microscope and a single serum sample is needed and the result is available in a few hours [53].

However, it has the disadvantage that these tests do not achieve the same sensitivity as a culture, since, unless specifically designed reagents are used, these tests cannot identify the serovars involved. The reagents for immunofluorescence should be prepared using anti-*Leptospira* sera with high levels of IgG, which are not commercially available. A negative result does not rule out infection, therefore, the results should be interpreted in conjunction with the results of the serological tests and it is necessary to analyze a second serum sample obtained in the convalescent phase to rule it out or confirm it by MAT [54].

This test was evaluated at the Colombian Institute of Tropical Medicine, obtaining a sensitivity of 89.47%, specificity of 100%, negative predictive value of 95.2% (95% CI 82.6–99.2) and positive predictive value of 100% [34]. Consequently, this technique represents an alternative diagnostic tool for leptospirosis and seroepidemiological studies [29,34].

### 3.5. Rapid Tests

Rapid tests for leptospirosis diagnosis consist of a lateral flow chromatographic immunoassay that detects a specific *Leptospira* antigen, aptamer, or other recognition molecule [55]. These tests have the advantage that results are obtained quickly (20 min) and do not require special equipment or training. The Standard Diagnostic Rapid Test is a qualitative assay that enables the differential detection of *Leptospira interrogans* IgG and/or IgM antibodies in human serum samples; however, this type of test provides only a preliminary result and should not be used as the sole criterion for diagnosing the disease [56,57].

Several rapid immunochromatographic tests have been developed to detect antibodies or biomarkers against *Leptospira* that offer ease of use, access, and affordability, but their diagnostic accuracy is not yet well established [56,57,58].

These are commercially available and exhibit adequate performance according to some authors [59]. For example, an evaluation of a test aimed at detecting a polysaccharide derived from the lipopolysaccharide (LPS) of non-pathogenic *Leptospira biflexa* serovar pathoc in samples from Korea, Bulgaria, and Argentina was carried out. The results showed a sensitivity of 93.9%, 100%, and 81.0%, respectively, and a specificity of 97.9%, 100%, and 95.4%, respectively [60]. However, other investigations have concluded that they are not adequate for the detection of acute leptospirosis in the population when evaluating their accuracy [35,57]. These investigations have reported an overall sensitivity ranging from 1.8% to 75% and a specificity ranging from 52.3% to 97.7% when evaluating five different rapid tests [57]. Similarly, these rapid tests have been poorly validated for Colombia [61].

### 3.6. Microagglutination (MAT)

MAT is a gold standard serological test for detecting antibodies to *Leptospira* [42], identifying the serovar causing the infection. It is based on the maintenance of live strains of *Leptospira* in culture, which makes it expensive and laborious, limiting its use to specialized laboratories, because they require highly qualified personnel and darkfield microscopy. This test not only identifies the serovar, but also the antibody titer, indicating whether the infection is recent. However, a single sample is inconclusive due to the persistence of antibodies in endemic areas. Culture of *Leptospira* is difficult and time-consuming, so serology is essential for diagnosis, and in some cases, although the specificity of MAT is good, significant serological cross-reactions between *Leptospira* serotypes and serogroups may occur [39].

To perform the microscopic agglutination test (MAT), it is essential to choose the right antigens. These antigens should encompass both strains representative of the serogroups present in the specific region, and those known to be prevalent in the host species under investigation in another geographic area are not always available [29].

The MAT test requires two serum samples taken in the acute phase and another in the convalescent phase 15 days apart and was standardized in Colombia in 2019 [62]. In tropical, low-income regions, access and adequate diagnosis is difficult when patients are required to return two weeks after hospitalization to submit a second sample [63]. Moreover, as noted, in these tropical and subtropical areas, other infectious agents circulate which have clinical manifestations that are very similar to leptospirosis, sometimes causing this differential diagnosis to be overlooked, delaying diagnosis and treatment, and in the worst cases, resulting in death [14].

### 3.7. Molecular Diagnostics

Several methods based on nucleic acid amplification (NAAT) have been developed for the early detection of *Leptospira* spp., ranging from conventional polymerase chain reaction (PCR) and its nested variant to real-time PCR and loop-mediated isothermal amplification (LAMP) [64,65]. In clinical samples, molecular detection is more sensitive than isolation and is the method with the greatest opportunity for early detection [66,67]. During the acute phase, from day five post-infection, spirochetes detectable by PCR circulate in the blood for up to 15 days at a concentration between 102 to 106 leptospires per mL, decreasing progressively [68]. For early diagnosis, samples of blood or serum are adequate [69], but a greater detection sensitivity has been observed using PCR of plasma samples obtained from blood anticoagulated with EDTA. Plasma or blood anticoagulated with heparin is less sensitive because it is a PCR inhibitor [70]. The sample can be taken up to two days after antibiotic therapy has been instituted [71].

The excretion of leptospires in urine occurs at the end of the acute phase [67], therefore it has no value for early diagnosis, but is useful for late phase detection, post mortem studies or detection in animal reservoirs [37,72]. However, inhibitors can be found in this type of sample [73]. *Leptospira* DNA has also been detected in cerebrospinal fluid, aqueous humor, and renal tissue [45,74].

The sensitivity of the amplification method is highly dependent on the extraction methods employed [73,75]. The reduction in the cost and availability of rapid extraction methods is a determining factor. Magnetic bead extraction allows for the concentration of molecules and the exclusion of most inhibitors, providing an increase in the analytical sensitivity.

Among the criteria evaluated for the selection of molecular targets are the genomic stability, selectivity in different sample types, inclusivity of pathogenic strains and exclusion capacity for saprophytes detected by common gene sequences coding for the 16S rRNA (rrs) region [24]. The detection of pathogenic species has been enabled through the design of fragments of enzyme sequences such as ligA [76] or gyrB [24], constitutive genes such as the translocase preprotein secY [24,77], and pathogenicity factors such as the outer membrane protein lipL32 [24,70,71,77], the latter being one of the most widely used targets due to its high sensitivity and specificity in real-time PCR assays [78].

Real-time PCR detection with probes or intercalants is more sensitive, timely, and less susceptible to contamination than the conventional variant (Table 1), detecting between 102 to 103 leptospires per mL and achieving an analytical sensitivity of 1 to 50 copies in acute phase serum or plasma samples [71]. Taking into account the different extraction methods used, it has been proposed that the presence of inhibitors in urine samples may affect the reproducibility, resulting in a lower detection limit [24,75]. Positive results can be obtained from samples containing as little as 10 copies [73].

Although quantitative detection by real-time PCR has been applied for the purpose of estimating the clinical stage, outcome, or predictive power, the performance characteristics of this approach do not differ much from qualitative methods and sometimes they are even better [77]. Some authors have established a threshold of 104 leptospires per mL for cases with pulmonary involvement and a fatal course [79], but this value differs from other findings where no correlation is observed, so it does not establish a precise criterion and its clinical usefulness is debatable [68].

LAMP is an alternative that allows for DNA amplification at a stable temperature, with detection systems based on turbidity or fluorescence, with analytical sensitivity between 2 and 100 leptospires per reaction, but questionable specificity. It has few requirements for extraction, but a processing time, equipment, and investment comparable with PCR [64,67,80]. Although validation processes must have the same rigor, for this reason there is no significant reduction in the final cost. One of the main advantages of this method may be the ability to run it at the point of care (POC) or in facilities that are not equipped with robust machines.

In general, despite the advantages described, few assays have been validated to the point of determining their diagnostic accuracy and some comparisons have determined a lower clinical sensitivity of molecular methods relative to MAT [73]. Particularly in Colombia, real-time PCR is not yet commercially available for the diagnosis of leptospirosis, so the need to standardize a test for the predominant serovars in the country is evident. This is because molecular methods offer performance characteristics that have been supported by sufficient scientific evidence to be considered an appropriate diagnostic tool for leptospirosis.

## 4. Leptospirosis in Colombia

Leptospirosis is endemic to Colombia. This status was declared in 2007 through the National Public Health Surveillance System (SIVIGILA) based on the Primary Data Generation Units (UPGD) and Reporting Units (UI) taken weekly throughout the country. The underreporting of this disease, however, is a reality. The purpose of this review is to highlight the importance of leptospirosis. While access to rapid diagnosis is available at practically all levels of care for dengue and malaria, leptospirosis—a doubly neglected disease—deserves recognition as a serious public health problem in Colombia. It is a preventable disease the treatment of which, apart from being easily accessible, is low-cost. As such, this study will examine leptospirosis diagnostic tools in order to determine which may be more adequate, timely, low-cost, easily accessible, and useful for public health surveillance and for the benefit of the affected persons.

**Figure 1:**
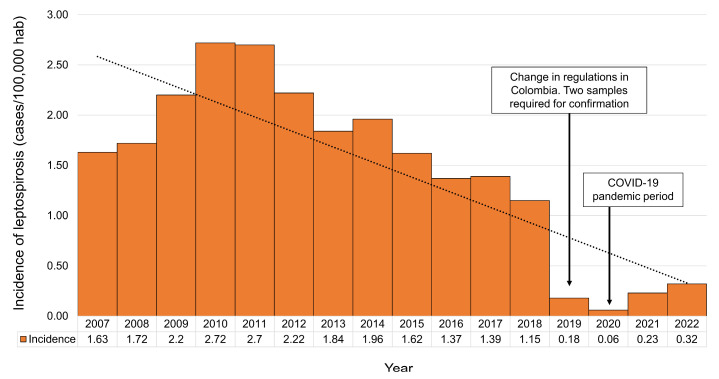
Incidence of leptospirosis per 100,000 inhabitants in Colombia, period 2007–2022, week 21. Adapted from: National Institute of Health (2022) [84].

In 2010, 2261 suspected cases of leptospirosis were reported by SIVIGILA, 71.9% of which corresponded to patients requiring hospitalization, and only 54.6% confirmed in a laboratory, giving an incidence of 2.71 per 100,000 inhabitants [81] (Figure 1). Since 2011, the National Institute of Health (INS) has reported that “there is currently routine notification to SIVIGILA, but despite this the underreporting of this event does not allow us to establish the current dimension of the disease in the country” [82]. The trend of incident cases in Colombia shows that between 2008 and 2012 there was an increase in the number of cases (Figure 1). However, leptospirosis case reporting changed as of 2019 [62], associated, to some extent, with the requirement for two paired sera: one in the acute phase and one in the convalescent phase for laboratory confirmation, which has possibly contributed to underreporting by laboratories [81].

A study of leptospirosis incidence and underreporting in Urabá recorded a cumulative incidence rate of 66.5 per 100,000 inhabitants for the period 2010–2012. The case fatality rate was 2.15% in the region, implying that leptospirosis morbidity and mortality are underreported by 27.8% and 66.6%, respectively when the data from the study were compared with those officially recorded by SIVIGILA. It is worth mentioning that the inclusion of a strain native to the Urabá region in the MAT panel increased the positivity by 15% [29].

This underreporting is also reflected in the reduction in reported cases and the decrease in incidence nationwide as of 2019 (Figure 1). In that year, only 467 of 2240 cases had access to paired tests for MAT, with 91 of these reported as positive and 376 as negative [62]. This was the second lowest incidence (0.18 cases per 100,000 population), after the incidence obtained in 2020 (Figure 1), where the higher underreporting was likely due to the COVID-19 pandemic that affected Colombia from March of that year. A similar phenomenon was observed in the surveillance systems of other parts of the world [83].

This downward trend is partly due to the fact that often, the patient does not return to the health center for a second sample collection. This makes laboratory confirmation impossible in accordance with the requirements established for the diagnosis of leptospirosis and the performance of MAT through the public health laboratory network [43]. From 2019, only paired sera from patients with a clinical suspicion of leptospirosis and those who had a positive ELISA result were processed by MAT, and were included in the surveillance system with the MAT results [62]. Researchers attributed the underreporting of cases to the lack of adequately equipped laboratories and trained personnel [85]. This contributes to the misdiagnosis of diseases that, like leptospirosis, present a variable symptomatology that confuses the differential diagnosis with other tropical diseases, in turn hindering confirmatory diagnosis in endemic areas.

On the other hand, in epidemiological week 15 of 2022, 2141 suspected cases of leptospirosis were reported that could not be confirmed due to the lack of paired tests with intervals of 10 to 15 days for MAT testing. Although many of these cases presented a positive ELISA test, this test is not considered confirmatory of the disease. In addition, the differential diagnosis of other diseases sharing symptoms with leptospirosis were not made due to the positive ELISA result obtained [81], this also contributes to the underreporting of other tropical diseases.

In Colombia, according to the Guidelines for Laboratory Surveillance of *Leptospira* spp., cases that raise the suspicion of leptospirosis require compliance with a diagnostic algorithm for laboratory confirmation, which consists of taking two samples, the first in the acute phase of the disease and the second in the convalescent phase (10 to 15 days after the first sample is taken). Initially, an indirect ELISA test is required in the samples of both phases of the disease, which must be later confirmed through the MAT technique, which is processed in the microbiology laboratory of the National Institute of Health of Colombia [43].

Other diagnostic methods are also used, such as blood culture (day 1–7 symptom onset) or urine (day 10–40 symptom onset) using the Ellinghausen–McCullough–Johnson–Harris (EMJH) medium. These cultures should be kept at a constant temperature of 30 ± 2 °C for a minimum period of 16 weeks, and ideally this incubation should be extended to 26 weeks. The time required to detect a positive culture may vary depending on the *Leptospira* serotype and the number of microorganisms present in the sample. Cultures should be examined regularly, every 1–2 weeks, using a high-quality darkfield microscope. This diagnostic method has very late results due to the incubation [43,50].

### 4.1. Statistical Calculations

Data collection: the data on the cases, deaths, and sociodemographic information of patients diagnosed with leptospirosis in Colombia from 2010 to 2021 were taken from the microdata base of the SIVIGILA website available at: http://portalsivigila.ins.gov.co/Paginas/Buscador.aspx# (accessed on 28 March 2023) [86]. These data were compiled in a single Excel file to perform calculations of the mortality rate, case fatality rate, YPLL, and WYL (Table S1). The population data such as the total population and life expectancy by sex and year in Colombia were taken from the web page http://datosmacro.com (accessed on 28 March 2023) [87], of which the source of data comes from the National Administrative Department of Statistics of Colombia (DANE).

Calculation of indicators: to calculate the Years of Potential Life Lost (YPLL) and Work Years Lost (WYL), twenty age groups were established, divided into four-year ranges, for example, group 1 includes all patients between 1 and 4 years and group 20 includes all patients between 95 and 99 years (Table S1).

The calculation of WYL was based on data on deaths per year. The life expectancy per year was established with respect to the data provided by http://datosmacro.com (accessed on 28 March 2023) [87] and the value of WYL was obtained from the multiplication of the difference between the maximum established, i.e., life expectancy, and the average of the ages of the patients who died in this period and the number of deaths reported for this period (Table S1) as indicated in the following formula [88].

Equation:

(1) $$AVPP=\sum_{i=l}L\left[\left(L-i\right)\times d_{I}\right],$$

l: lower age limit established

L: the established upper age limit i: the age of death

di: the number of deaths at age i

The WYL quantifies the work time lost due to death before or during the productive age [14], therefore, the age limit was established as the ages pre-established in article 33 of Law 100 of 1993 until 2014, and in article 36 of the same law for 2014 onwards. The WYL value per year was obtained from the sum of all the values greater than zero as a result of the subtraction of the productive age determined and the age at death of each patient (Table S1).

All calculations and data used are available in the Supplementary Materials.

Some socioeconomic and health indicators regarding leptospirosis in Colombia point to a decrease in the quality of life of people with this disease. Indeed, leptospirosis accounts for 2% of the premature deaths each year in Colombia. In addition, it is also responsible for some economic loss, as an estimated maximum of 648 lost work years for 2012 and a minimum of 46 lost work years for 2020 are attributable to premature death, since it is estimated around 2 years of life are potentially lost per 100,000 inhabitants in Colombia (Table 2) [86,87]. This represents a considerable economic loss, assuming that the latter is an underestimate due to the increased underreporting resulting from the changing diagnostic standards and the effects of COVID-19 policy that year. Furthermore, this year there was the highest percentage of the fatality rate, suggesting that the cases reported to the surveillance system were only those in serious conditions, contributing to the underreporting this year (Table 2).

In this sense, the main weakness in the surveillance of the disease is the lack of diagnostic documentation, resulting in inadequate clinical management, diagnosis, and treatment, as well as the poor access to effective, specific, fast, affordable, and widely available diagnostic tests [14,74]. Molecular tests are an alternative that can guide the treatment of the patient and consequently the timeliness of the case reporting to SIVIGILA. This would encourage a reduction in the underreporting of the disease [89].

The strengthening of the technological infrastructure derived from the SARS-CoV-2 pandemic solves, to a large extent, the disadvantage of requiring appropriate facilities for molecular diagnostics. A network of 227 laboratories currently installed and distributed throughout the national territory has the capacity for PCR testing (https://www.ins.gov.co/Noticias/Paginas/coronavirus-laboratorios.aspx (accessed on 28 March 2023)) [90]. The possibility of applying the method for pathogen detection in animals or environmental samples can be used in a preventive public health approach to avoid potential outbreaks.

### 4.2. Regulatory Framework

The authorization of methods for human diagnosis in Colombia is carried out by the Instituto de Vigilancia de Medicamentos (INVIMA) [91]. The surveillance and control of laboratories is carried out by the Departmental Health secretaries. When laboratories are verified to have met the requirements (established in the [92,93]), y are permitted to test with registered kits; for accredited laboratories, the implementation of technical quality standards is required [94]. In animal health, the regulation is carried out by the Colombian Agricultural Institute and registered laboratories [95] must perform accreditation processes to be authorized to perform tests on large species and production animals.

## 5. Proposal to Improve the Diagnosis of Leptospirosis in Colombia

In 2013, during the third meeting of the Global Leptospirosis Environmental Action Network (GLEAN), it was concluded that the use of real-time PCR could be a feasible alternative for the accurate and rapid diagnosis of leptospirosis. However, the need to improve the cost–benefit ratio in order to generate early registration in the epidemiological centers of each country was stressed [96].

Real-time PCR is not yet commercially available for the diagnosis of leptospirosis in Colombia; however, it is estimated that the price of “in house” tests is approximately COP 250,000 (USD 60.00). In addition, it has other advantages that position it as a better option for leptospirosis diagnosis, as it can be carried out on blood and tissue samples (Table 1). This permits quick results, and sensitive and specific diagnosis in the acute phase of the disease, since it can give a positive result from blood before a detectable antibody titer has developed [97,98]. It does not require paired serum samples and, depending on primer design, could include the detection of several circulating species in the country (Table 1).

On the other hand, conventional PCR in Colombia has a commercial cost of around COP 180,000 (USD 45.00) (March 2023), and has a sensitivity of 100% (95% CI: 97.9–100%) in blood samples within the first seven days of the disease [99], compared to 71.1% for MAT [100] (Table 1).

Considering not only the advantages that these molecular techniques provide to the diagnosis of leptospirosis but also taking into account Colombia’s response to the SARS-CoV-2 pandemic, which facilitated the development and provision of 227 laboratories capable of performing real-time PCR tests, located in 28 of the 32 departments of the country [90], the standardization of a molecular test that allows the specific diagnosis of serovars and species of *Leptospira* in the country is proposed. This initiative would support the diagnosis of leptospirosis in its acute phase [101], potentially reducing the underreporting of morbidity and mortality due to leptospirosis in Colombia. In addition, it would contribute to the reduction in the costs and time spent on laboratory diagnostic tests, contribute to a more accurate diagnosis of the disease, support its management by healthcare personnel, make optimal use of antibiotics, and reduce patient complications and hospital stays.

## 6. Conclusions

The underreporting of the incidence of leptospirosis has repercussions at the local and national level for the epidemiological surveillance system. Additionally, there are economic consequences due to hospitalization, the days of incapacity of those affected, and the years of work lost due to premature deaths, not to mention the indiscriminate use of antibiotics with the consequent increase in bacterial resistance in patients who consult the hospital network with acute febrile syndrome of unknown etiology.

Based on the evidence presented in this review, it is suggested to consider the PCR test as the national reference diagnosis for leptospirosis in the acute phase of the disease. This test is specific, sensitive, and rapid, which allows for timely treatment and the generation of information that will improve the evaluation criteria for leptospirosis surveil-lance, especially in terms of diagnostic and morbimortality indicators that are better adjusted to the reality of the country. The inclusion of this test in the country’s surveillance laboratories is facilitated by the fact that Colombia has trained personnel and a network of laboratories capable of processing it, and this would only require consensus on the conditions of the validation and standardization of the test. However, the importance of the microscopic agglutination test (MAT) is recognized as an epidemiological reference serological test to identify the causal serogroup of the disease. The combination of both tests, PCR and MAT, remains relevant to support epidemiological surveillance, strengthening diagnostic confirmation and knowledge about leptospirosis in Colombia, including key information such as reservoirs, circulating strains, seasonal and geographic patterns, and populations at risk, which will allow for the implementation of appropriate prevention and intervention strategies.

## Figures and Tables

**Table 1:** Price (2023), advantages, and disadvantages of the tests used for the diagnosis of leptospirosis in Colombia.

Test	Price (COP/US)	Advantages	Disadvantages
ELISA	$80,000 */20	- Economic. - Does not require a highly complex laboratory. - Highly sensitive in the convalescent phase of the disease.	- Requires paired samples - Difficult to obtain the second sample in patients. - It can present false negatives in the acute phases of the disease, when antibodies have not yet been generated (low sensitivity).
MAT	$438,000/105	- Determines the specific serogroup. - Highly sensitive if isolated serovars circulating within the study area are included.	- High price. - Requires paired samples. - Access to results in an untimely manner. - Few serovars available; however, the sensitivity increases when a greater number of strains are included in the evaluation panel. - Cross reaction that decreases its specificity.
Conventional PCR	$180,000 **/45	- Fast. - Does not require paired samples. - Highly sensitive and specific in the acute phase of the disease when blood or serum samples are taken. - Depending on the primer design, gender can be determined.	- High cost that could be neutralized as the number of tests increases. - Requires specialized equipment, specific and restricted laboratory access infrastructure, as well as highly qualified personnel. - It can generate false negative results in urine samples due to the presence of inhibitors.
Real-time PCR	$250,000 **/60	- Fast. - Does not require paired samples. - Highly sensitive and specific in the acute phase of the disease when blood or serum samples are taken. - Depending on the primer design, gender can be determined. - It can provide information about the concentration of the bacteria in the sample if a standardization curve is made.	- High cost that could be neutralized as the number of tests increases. - Requires specialized equipment and a dedicated laboratory space, as well as highly qualified personnel. - It can generate false negative results in urine samples due to the presence of inhibitors.

* Test price in 2022. ** Approximate cost based on separate reagent purchase. There is no commercial kit registered with INVIMA for the year 2022.

**Table 2:** Mortality rate, case fatality rate, Years of Life Potentially Lost (YLPP), YLLP index, and Years of Work Lost (WYL) due to leptospirosis from 2010 to 2021 in Colombia.

Year	National Death Rate per 100,000	Case Fatality Rate (%)	YLPP	YLPP Index	WYL
2010	0.074	2.67	1022.86	2.32	616
2011	0.072	2.58	848.12	1.90	434
2012	0.056	2.40	1059	2.35	648
2013	0.051	2.65	537.53	1.18	253
2014	0.059	2.88	965.64	2.10	646
2015	0.028	1.66	408.89	0.88	215
2016	0.034	2.40	429.68	0.91	204
2017	0.027	1.89	527.09	1.11	335
2018	0.012	1.06	231.66	0.48	133
2019	0.006	3.37	97.87	0.19	57
2020	0.004	6.25	72.92	0.14	46
2021	0.010	4.27	- *	- *	77

* Life expectancy data for the year 2021 are not yet available to calculate YLPP and YLPP index. Source: Own elaboration based on National SIVIGILA Statistics [86] and population data available in the expansion datosmacro.com (accessed on 13 April 2023) [87].

## Data Availability

All demographic data used to calculate the rates and rates shown in this review article are found in: http://portalsivigila.ins.gov.co/Paginas/Buscador.aspx# (accessed on 28 March 2023) [86] and http://datosmacro.com (accessed on 13 April 2023) [87]. In addition, you can find in detail the data compiled and the calculations performed to calculate the rates, Work Years Lost, and Years of potential life lost in the Supplementary Materials Tables S1–S4.

## Supplementary Materials

The following supporting information can be downloaded at https://www.mdpi.com/article/10.3390/microorganisms11112759/s1, Table S1: Age groups, Table S2. Data used to calculate rates, Table S3. Data and calculation of Work Years Lost (WYL), Table S4. Data for the calculation of Years of potential life lost (YPLL).
